# Retraction of: Survival after pentobarbitone overdose confirmed through Prescription, Recreational and Illicit Substance Evaluation (PRISE) programme in Australia

**DOI:** 10.1093/fsr/owad023

**Published:** 2023-08-08

This is a retraction of: Thanjira Jiranantakan and others, Survival after pentobarbitone overdose confirmed through Prescription, Recreational and Illicit Substance Evaluation (PRISE) programme in Australia, *Forensic Sciences Research*, Volume 6, Issue 3, September 2021, Pages 231–236, https://doi.org/10.1080/20961790.2021.1975613

Due to the sensitive nature of this topic and the associated health and safety risks, this case report is being retracted and the content has been removed, both at the request of the authors and with the agreement of the editors.

